# Factors Associated with Inadequate Intravenous Colistin Dosages: Post Hoc Analysis of a Multicenter, Cross-Sectional Study

**DOI:** 10.3390/antibiotics10121554

**Published:** 2021-12-19

**Authors:** Daniele Roberto Giacobbe, Michele Mirabella, Matteo Rinaldi, Angela Raffaella Losito, Francesca Raffaelli, Filippo Del Puente, Carolina Saffioti, Malgorzata Mikulska, Maddalena Giannella, Pierluigi Viale, Mario Tumbarello, Matteo Bassetti, on behalf of SITA GIOVANI (Young Investigators Group of the Società Italiana Terapia Antinfettiva) and the COLI-CROSS Study Group

**Affiliations:** 1 Department of Health Sciences (DISSAL), University of Genoa, 16132 Genoa, Italy; filippo.del.puente@galliera.it (F.D.P.); m.mikulska@unige.it (M.M.); matteo.bassetti@unige.it (M.B.); 2 Clinica Malattie Infettive, IRCCS Ospedale Policlinico San Martino, 16132 Genoa, Italy; michelemirabella@hsanmartino.it; 3 Operative Unit of Infectious Diseases, IRCCS Azienda Ospedaliero-Universitaria di Bologna, 40138 Bologna, Italy; mat.rinaldi1989@gmail.com (M.R.); maddalena.giannella@unibo.it (M.G.); pierluigi.viale@unibo.it (P.V.); 4 Dipartimento di Scienze di Laboratorio e Infettivologiche, Fondazione Policlinico Universitario A. Gemelli IRCCS, 00168 Roma, Italy; lositoraffaella@yahoo.it (A.R.L.); franraffaelli@gmail.com (F.R.); 5 Department of Infectious Diseases, Galliera Hospital, 16128 Genoa, Italy; 6 Infectious Diseases Unit, IRCCS Istituto Giannina Gaslini, 16147 Genoa, Italy; carolinasaffioti@gaslini.org; 7 Department of Medical Biotechnologies, University of Siena, 53100 Siena, Italy; mario.tumbarello@unisi.it

**Keywords:** colistin, dosage, polymyxin, antimicrobial stewardship, acute kidney injury

## Abstract

Colistin is a last-resort agent for the treatment of infections due to Gram-negative bacteria with difficult-to-treat resistance. The primary objective of this post hoc analysis of a cross-sectional study conducted in 22 Italian hospitals was to assess factors associated with inadequate intravenous colistin dosage. Overall, 187 patients receiving intravenous colistin were included in the analyses. Inadequate colistin dosages were administered in 27% of cases (50/187). In multivariable analysis, AKI (dummy variable with KDIGO stage 0 as a reference, odds ratio (OR) 3.98 with 95% confidence interval (CI) 1.48–10.74 for stage 1, OR 4.44 with 95% CI 1.17–16.93 for stage 2, OR 9.41 with 95% CI 1.59–55.70 for stage 3; overall *p* = 0.001) retained an independent association with inadequate colistin dosage, whereas the presence of a central venous catheter was associated with adequate colistin dosage (OR: 0.34 for inadequate dosage, 95% CI: 0.16–0.72, *p* = 0.004). These results were confirmed in an additional multivariable model with the center as a random effect. The association between AKI and inadequate dosage may reflect the perception of an increased risk of nephrotoxicity in patients with impaired renal function, which nonetheless should not be accompanied by dosage reductions beyond those recommended and could represent the target of dedicated antimicrobial stewardship efforts.

## 1. Introduction

Colistin is a last-resort agent for the treatment of infections due to Gram-negative bacteria with difficult-to-treat resistance, especially carbapenem-resistant *Acinetobacter baumannii*, carbapenem-resistant Enterobacterales, and carbapenem-resistant *Pseudomonas aeruginosa* [1,2,3,4].

Although an important reduction in colistin use has been registered in the past two years, owing to the availability of novel, less nephrotoxic, and possibly more efficacious beta-lactams and beta-lactam/beta-lactamase inhibitor combinations, in selected cases (e.g., allergy or resistance to novel agents), colistin may still represent a precious last-resort treatment option for infections due to carbapenem-resistant Gram-negative bacteria; thus, our efforts to optimize its use and efficacy in real-world practice should still not be abandoned [4,5,6,7,8,9,10].

In the present post hoc analysis of a previous large multicenter study describing the use of colistin in Italian hospitals [11], we aimed to identify modifiable predictors of inadequate intravenous colistin dosage that could represent the selected target of future dedicated antimicrobial stewardship interventions.

## 2. Methods

This is a post hoc analysis of a multicenter, cross-sectional study describing colistin use in 22 Italian hospitals (COLI-CROSS study) [11]. The primary objective of this post hoc analysis was to assess factors associated with inadequate intravenous colistin dosage. All patients included in the main study who received intravenous colistin were included. In line with the lack of detailed dosage information in patients receiving hemodialysis in the COLI-CROSS study, patients on hemodialysis were excluded from this post hoc analysis. Inadequate colistin dosage was defined as the absence of an adequate loading dose (i.e., 9 million units of colistimethate) and/or the absence of adequate maintenance dosages according to the Committee for Medicinal Products for Human Use of the European Medicines Agency, adjusted to renal function (see supplementary material) [12]. The COLI-CROSS study was approved by the Ethics Committee of the coordinating center (Liguria Region Ethics Committee, registry number 321/2017). The other participating centers followed the local ethical requirements. The study was conducted according to the guidelines of the Declaration of Helsinki.

### Statistical Analysis

The primary study analysis was the identification of factors associated with inadequate colistin dosage. To this aim, demographic and clinical variables were tested for their association with inadequate colistin dosage in univariable logistic regression models. Then, variables potentially associated with inadequate dosage in univariable models (*p* < 0.10) were included in an initial multivariable logistic regression model, and further selected for the final model (model A) using a stepwise backward procedure. The variables included in model A were additionally tested for their association with inadequate colistin dosage in a generalized, linear mixed model (model B, with logit as the link function and center as a random effect). The analyses were performed using R Statistical Software version 3.5.2 (R Foundation for Statistical Computing, Vienna, Austria). The mixed model was built with the glmer function in the lme4 package for R Statistical Software.

## 3. Results

**Figure 1:**
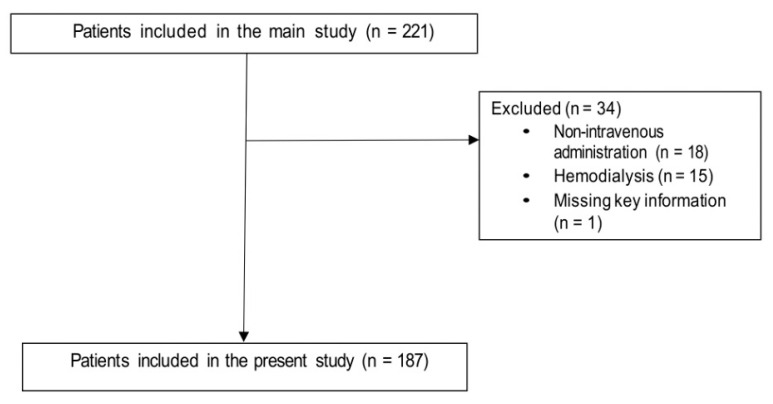
Flow-chart of the patient selection process.

Overall, 187 patients were included in the analysis (Figure 1). The first two columns of Table 1 show the demographic and clinical characteristics of patients who received adequate colistin dosages and those who receive inadequate colistin dosages. Overall, 50/187 patients (27%) received inadequate colistin dosages (lower than those recommended). Table 1 also shows the results of the univariable analysis of factors potentially associated with the administration of an inadequate colistin dosage. Increasing age, chronic renal failure, and acute kidney injury (AKI) were associated with inadequate colistin dosage in univariable comparisons, whereas presence of a central venous catheter was associated with adequate colistin dosage.

The results of the multivariable analysis of factors associated with inadequate colistin dosages are shown in Table 2. AKI (dummy variable with KDIGO stage 0 as reference, odds ratio [OR] 3.98 with 95% confidence intervals [CI] 1.48–10.74 for stage 1, OR 4.44 with 95% CI 1.17–16.93 for stage 2, OR 9.41 with 95% CI 1.59–55.70 for stage 3; overall *p* for the dummy variable 0.001) retained an independent association with inadequate colistin dosages, whereas presence of a central venous catheter was independently associated with adequate colistin dosages (OR 0.34 for inadequate dosages, 95% CI 0.16–0.72, *p* = 0.004). These results were confirmed in the additional multivariable model with the center as a random effect (model B, also shown in Table 2).

## 4. Discussion

The COLI-CROSS study overall registered high rates of adequate colistin dosages (79% for loading dosages and 85% for maintenance dosages, respectively) [11]. Nonetheless, a nonnegligible proportion of patients (21%) still did not receive a proper loading dose, a fact that prompted the conduction of the present post hoc analysis. Indeed, the identification of possible modifiable independent predictors of inadequate dosages could prompt dedicated antimicrobial stewardship interventions.

In our analysis, two factors showed an independent association with either inadequateness or adequateness of intravenous colistin dosages. On the hand, AKI was associated with an increased risk of receiving inadequate colistin dosages. In this regard, while it is well known that colistin is nephrotoxic, the perceived risk of nephrotoxicity should not lead clinicians to further reduce colistin dosages beyond the standard adjustments recommended by guidelines since this could unacceptably increase the risk of suboptimal colistin concentrations at the site of infection, especially for pneumonia [4,8,13]. On the other hand, the presence of a central venous catheter was independently associated with adequate colistin dosage in our analysis. The exact reason for this potential protective effect remains somewhat elusive, although some possible non-mutually exclusive explanations are the following: (i) difficulty to administer all required doses in some patients without a central catheter and concomitant difficult peripheral venous access; (ii) the presence of a higher proportion of patients with a central venous catheter in intensive care units, where infections by carbapenem-resistant Gram-negative bacteria may be more frequent, and consequently, there could more frequent and confident administration of adequate colistin dosages. In this regard, it is of note that, although not statistically significant, the direction of the association between prescription of by infectious diseases specialists and adequateness of colistin dosages was towards improved adequateness, that may support the need of expertise in prescribing this last resort agent.

The present study has some important limitations. Besides the inability to assess the adequateness of dosages in peculiar populations such as in patients on hemodialysis owing to the lack of detailed information (see study methods), it is of note that we assessed the adequateness of dosages based on the standards at the time of the study, whereas the international consensus guidelines on the optimal use of the polymyxins released subsequently suggest the possible need of further increasing manteinance dosages in patients with augmented renal clearance [4]. Finally, the lack of therapeutic drug monitoring is an inherent limitation of most large real-life studies on colistin use since it is usually limited to few hospitals and laboratories [13].

In conclusion, we found an association between AKI and inadequate dosage that may reflect the perception of an increased risk of nephrotoxicity in patients with impaired renal function, which nonetheless should not be accompanied by dosage reductions beyond those recommended and could represent the target of dedicated antimicrobial stewardship efforts, together with the proper administration of all required colistin doses.

## Figures and Tables

**Table 1:** Characteristics of the study population and univariable analysis of factors associated with inadequate intravenous colistin dosage.

Variable	Patients Receiving Inadequate Dosage (%) (*n* = 50)	Patients Receiving Adequate Dosage (%) (*n* = 137)	OR (95% CI)	*p*
Age in years, median IQR	69 (53–80)	61 (48–71)	1.03 (1.00–1.05)	0.021
Male gender	32 (64)	81 (59)	1.23 (0.63–2.40)	0.547
Previous treatment with colistin	8 (16)	20 (15)	1.11 (0.46–2.72)	0.812
Hospital stay before colistin initiation in days, median (IQR)	17 (4–40)	23 (13–50)	1.00 (0.99–1.01)	0.960
ICU stay	22 (44)	56 (41)	1.13 (0.59–2.19)	0.701
Diabetes mellitus	15 (30)	27 (20)	1.75 (0.84–3.65)	0.138
Chronic renal failure	5 (10)	3 (2)	4.96 (1.14–21.60)	0.033
Solid neoplasm	10 (20)	23 (17)	1.24 (0.54–2.83)	0.611
Hematological malignancy	2 (4)	13 (9)	0.40 (0.09–1.83)	0.236
Charlson score, median (IQR)	2 (1–3)	2 (1–3)	1.12 (0.97–1.29)	0.136
Presence of CVC	30 (60)	109 (80)	0.39 (0.19–0.78)	0.008
Presence of urinary catheter	39 (78)	113 (82)	0.75 (0.34–1.68)	0.488
Mechanical ventilation	18 (36)	37 (27)	1.52 (0.76–3.03)	0.234
Neutropenia	2 (4)	11 (8)	0.48 (0.10–2.23)	0.347
Septic shock	12 (24)	27 (20)	1.29 (0.59–2.79)	0.523
Pulmonary infection	15 (30)	44 (32)	0.91 (0.45–1.83)	0.783
KDIGO stage of AKI				0.002
No AKI	31 (62)	120 (88)	(ref)	
Stage 1	10 (20)	10 (7)	3.87 (1.48–10.12)	
Stage 2	5 (10)	5 (4)	3.87 (1.05–14.22)	
Stage 3	4 (8)	2 (1)	7.74 (1.36–44.23)	
Type of prescriber ^§^				0.346
Infectious diseases specialist	36 (72)	109 (81)	(ref)	
Intensive care specialist	10 (20)	16 (12)	1.89 (0.79–4.54)	
Others ^§§^	4 (8)	9 (7)	1.35 (0.39–4.63)	
Targeted therapy ^§§§^	40 (80)	101 (74)	1.43 (0.65–3.14)	0.379
Combination therapy	42 (84)	108 (79)	1.41 (0.60–3.33)	0.434

Results are presented as n (%) unless otherwise indicated. AKI, acute kidney injury; CI, confidence intervals; CVC, central venous catheter; ICU, intensive care unit; IQR, interquartile range; KDIGO, Kidney Disease: Improving Global Outcomes; OR, odds ratio. ^§^ Information missing for 3/187 patients (2%). ^§§^ Hematologist (n = 7), pneumologist (n = 3), internist (n = 1), neurologist (n = 1), surgeon (n = 1). ^§§§^ With regard to carbapenem-resistant *Acinetobacter baumannii* (CRAB), carbapenem-resistant Enterobacterales (CRE), and carbapenem-resistant *Pseudomonas* aeruginosa (CRPA): CRAB (n = 60), CRE (n = 38), CRPA (n = 24), CRAB plus CRE (n = 11), CRE plus CRPA (n = 5), CRAB plus CRPA (n = 2), CRAB plus CRE plus CRPA (n = 1).

**Table 2:** Multivariable analysis of factors associated with inadequate intravenous colistin dosage *.

**Model A (AIC 204.5)**	**OR (95% CI)**	***p***
Presence of CVC	0.34 (0.16–0.72)	0.004 ^§^
KDIGO stage of AKI		0.001 ^§^
No AKI	(ref)	
Stage 1	3.98 (1.48–10.74)	
Stage 2	4.44 (1.17–16.93)	
Stage 3	9.41 (1.59–55.70)	
Model B ** (AIC 199.4)	**OR (95% CI)**	***p***
Presence of CVC	0.33 (0.15–0.75)	0.008 ^§^
KDIGO stage of AKI		0.002 ^§^
No AKI	(ref)	
Stage 1	4.73 (1.56–14.37)	
Stage 2	4.83 (105–22.22)	
Stage 3	8.23 (1.20–56.64)	

AIC, Akaike information criterion; AKI, acute kidney injury; CI, confidence intervals; CVC, central venous catheter; OR, odds ratio. * Only variables retained in the final multivariable model after stepwise backward selection are shown in the table. ** Model B included center as a random intercept (variance 0.696, standard deviation 0.834). ^§^ *p* < 0.05.

## Data Availability

The data presented in this study are available on reasonable request from the corresponding author.

## Supplementary Materials

The following are available online at https://www.mdpi.com/article/10.3390/antibiotics10121554/s1, File S1: adequate colistin dosage in patients not undergoing hemodialysis according to recommendations at the time of the study.
