# Supplementary material for: Factors Associated with Inadequate Intravenous Colistin Dosages: Post Hoc Analysis of a Multicenter, Cross-Sectional Study
Source: Antibiotics (Basel). 2021 Dec 19;10(12):1554. doi: 10.3390/antibiotics10121554 (PMC8698974; doi:10.3390/antibiotics10121554)
Supplement: Supplementary file 1 [file antibiotics-10-01554-s001.zip › antibiotics-1509429-SI.pdf]

Adequate colistin dosage in patients not undergoing hemodialysis according to recommendations at the time of the study* [1]

Loading dose:

- 9 million international units (MIU)

Manteinance dosage (daily, divided in 2-3 doses) according to creatinine clearance (CrCL):

- CrCL ≥50 ml/min: 9 MIU
- CrCL <50-30 ml/min: 5.5-7.5 MIU
- CrCL <30-10 ml/min: 4.5-5.5 MIU
- CrCL <10 ml/min: 3.5 MIU

* The study was conducted before the release of the International Guidelines for the Optimal Use of the Polymyxins [2]

References

1. European Medicines Agency completes review of polymyxin-based medicines. Recommendations issued for safe use in patients with serious infections resistant to standard antibiotics. Annex III [last accessed 21 Nov 2021]. https://www.ema.europa.eu/documents/referral/polymyxin-article-31-referral-annex-iii_en.pdf.
2. Tsuji, B.T.; Pogue, J.M.; Zavascki, A.P.; Paul, M.; Daikos, G.L.; Forrest, A.; Giacobbe, D.R.; Viscoli, C.; Giamarellou, H.; Karaiskos, I., et al. International Consensus Guidelines for the Optimal Use of the Polymyxins: Endorsed by the American College of Clinical Pharmacy (ACCP), European Society of Clinical Microbiology and Infectious Diseases (ESCMID), Infectious Diseases Society of America (IDSA), International Society for Anti-infective Pharmacology (ISAP), Society of Critical Care Medicine (SCCM), and Society of Infectious Diseases Pharmacists (SIDP). *Pharmacotherapy* **2019**, *39*, 10-39, doi:10.1002/phar.2209.
